# Ethische Aspekte in der partizipativen Forschung – Reflexion von Herausforderungen und möglichen Beeinträchtigungen für Teilnehmende

**DOI:** 10.1007/s00103-020-03270-0

**Published:** 2020-12-30

**Authors:** Ina Schaefer, Petra Narimani

**Affiliations:** 1 Alice Salomon Hochschule Berlin, Alice-Salomon-Platz 5, 12627 Berlin, Deutschland; 2 Katholische Hochschule für Sozialwesen Berlin (KHSB), Köpenicker Allee 39, 10318 Berlin, Deutschland

**Keywords:** Partizipative Gesundheitsforschung, Prozessreflexion, Ethik, Schadensminimierung, Participatory health research, Process reflection, Ethics, Protection from harm

## Abstract

In der medizinischen Forschung hat sich die Auseinandersetzung mit ethischen Fragestellungen verbindlich etabliert. Dabei werden in der Regel die Kriterien *informiertes Einverständnis und Freiwilligkeit, Schadensminimierung sowie Vertraulichkeit und Anonymisierung* zugrunde gelegt. Auch in der sozialwissenschaftlichen Forschung und ebenso in der in Deutschland noch wenig verbreiteten partizipativen Forschung gewinnt die Auseinandersetzung mit ethischen Fragen an Bedeutung. Durch die aus dem Anspruch auf eine gleichberechtigte Zusammenarbeit resultierenden besonderen Beziehungen stellen sich ethische Fragen in der partizipativen Forschung anders als in der nicht partizipativen Forschung. In diesem Beitrag wird im Rahmen einer u. a. auf eine Dokumentenanalyse gestützten Prozessreflexion anhand eines Fallbeispiels aus der partizipativen Gesundheitsforschung illustriert, was genau unter Schadensminimierung verstanden werden kann. Das Fallbeispiel stellt dies aus Sicht der Wissenschaft wie der Forschenden aus der Lebenswelt dar.

Die Reflexion zeigt, welche Herausforderungen in der partizipativen Zusammenarbeit vor allem aus Sicht der Forschenden aus der Lebenswelt im partizipativen Prozess zu Beeinträchtigungen und damit zu einem Schadensrisiko führen können. Dabei geht es z. B. um Enttäuschungen, wenn dem Wunsch nach einer individuellen Unterstützung in einem Forschungsprozess nicht ausreichend gefolgt werden kann.

Aus den gesammelten Erfahrungen werden Schlussfolgerungen für den Umgang mit ethischen Aspekten in partizipativen Forschungsprojekten abgeleitet. Außerdem wird aufgezeigt, wie die Verknüpfung mit dem Diskurs in der nicht partizipativen qualitativen Forschung für eine Stärkung der ethischen Auseinandersetzung im partizipativen Prozess genutzt werden kann.

## Abstract (en)

In medical research, it is established practice to discuss ethical issues, usually based on the criteria *informed consent and voluntary participation, protection from harm, confidentiality, and anonymity*. The discussion of ethical questions is also gaining importance in social science research. The same applies to participatory research, which is not yet very widespread in Germany. Because this approach is based on equality between all those involved, it results in relationships uncommon in nonparticipatory research. Therefore, ethical questions are raised in a different way. Partly based on an analysis of documents, a case study from participatory health research will be used to illustrate how the principle of harm reduction can be recognized. The case study shows this from the perspective of both the researchers and the peer-researchers.

From the peer-researchers’ perspective, there are different challenges in participatory teamwork, which can bring impairments and consequently result in a risk of harm. This can lead to disappointments, for example, when from the peer-researchers’ perspective individual support during a research process is insufficient.

Conclusions for dealing with ethical aspects in participatory research projects will be derived from the gathered experiences. The application of the criteria used in nonparticipatory research can be an opportunity to strengthen the discussion of ethical issues as part of participatory processes. It can also be a chance to combine the ethical discourse in participatory and qualitative social science research.

## Einleitung

Ethik kann als die wissenschaftliche Auseinandersetzung mit *Moral* bzw. als eine Untersuchung der Moral verstanden werden. Dabei umschreibt *Moral* das System von Werten, Normen und Tugenden, das Maßstab für die Interaktion von Individuen oder sozialen Gruppen bis hin zur gesamten Gesellschaft ist [1]. Je nach Handlungsbereich können verschiedene Bereichsethiken unterschieden werden, so die *Ethik in den Gesundheitswissenschaften* und hier wiederum die Disziplin der *Forschungsethik*, die sich mit der Bewertung gesundheits- (oder auch krankheits-) bezogener Forschung befasst [1]. Im Mittelpunkt der Forschungsethik in den Sozial- und Humanwissenschaften „stehen der verantwortungsvolle Umgang mit menschlichen und tierischen Untersuchungsteilnehmenden und ihr Schutz vor unnötigen oder unverhältnismäßigen Beeinträchtigungen durch den Forschungsprozess“ [2, S. 163]. Verankert ist die Forschungsethik in den im Grundgesetz aufgeführten Grundrechten der Menschen und in der UN-Menschenrechtskonvention von 1950 [3].

In den verschiedenen Gebieten der Sozial- und Humanwissenschaften und auch in der partizipativen Gesundheitsforschung gibt es bezüglich der Forschungsethik keine einheitlichen Anforderungen [4]. Zugleich ist bestehenden rechtlichen Regelungen, die sich v. a. auf forschungsethische Gesichtspunkte beziehen, Rechnung zu tragen [5]. Für eine Reflexion forschungsethischer Herausforderungen in diesem Feld sind daher ethische Anforderungen verschiedener Professionen in den Sozial- und Gesundheitswissenschaften ebenso wie in der partizipativen (Gesundheits‑)Forschung diskutierte Anforderungen in den Blick zu nehmen. Eine Eingrenzung lässt sich durch den Bezug auf die in der partizipativen Forschung bisher dominierenden qualitativen Forschungsansätze vornehmen [6].

Die von Hopf benannte Auseinandersetzung der Beziehung zwischen Forschenden und Beforschten als Bestandteil von Forschungsethik in der qualitativen Sozialforschung ist auch essenzielles Kennzeichen partizipativer Forschung [5]; denn die Menschen, deren Lebensbereiche erforscht werden, gestalten die Forschung mit [7]. Insofern muss der Blick auf andere Professionen und auch auf die ethischen Vorstellungen der Adressat*innen-Gruppen gerichtet werden, für die die Beziehung zu den Beteiligten ebenfalls eine (teilweise hohe) Bedeutung hat.

## Ethische Anforderungen in der Gesundheitsforschung

Gegenüber den anderen Fachdisziplinen ist die forschungsethische Diskussion in der Medizin und der Psychologie – auch aus (leidvollen) historischen Gründen – besonders weit fortgeschritten [2, 6]. Großen Einfluss hat die Deklaration von Helsinki des Weltärztebundes, die einen Ordnungsrahmen für Forschung am Menschen, aber auch an identifizierbaren menschlichen Daten bildet [4]. Diese nimmt Bezug auf die bereits von Beauchamp und Childress identifizierten Prinzipien [8]:

- Freiwilligkeit und informierte Einwilligung,
- Schadensminimierung sowie Fürsorge,
- Anonymisierung und Vertraulichkeit der Daten.

In Hinblick auf den Fokus des Beitrags nehmen wir im Folgenden den Aspekt der Schadensminimierung in den Blick.

### Ethische Anforderungen in der human- und sozialwissenschaftlichen Gesundheitsforschung

In der sozialwissenschaftlichen Forschung findet in Bezug auf die oben angesprochenen Grundprinzipien zum Teil eine breitere Auseinandersetzung mit forschungsethischen Fragen statt, das Feld ist aber noch wenig systematisiert [6]. Institutionalisierte Prüfverfahren sind hier bislang nicht die Regel, auch wird dieses Vorgehen kritisch diskutiert [9]. Allerdings werden die zuvor genannten Prinzipien in nahezu allen Fachdisziplinen als Teil bereichsspezifischer ethischer Kodexe herangezogen [2]. In Bezug auf ihre Umsetzbarkeit werden hier insbesondere das informierte Einverständnis sowie die Vertraulichkeit der Daten unter Hinweis auf bestehende gesetzliche Regelungen beschrieben.

In den Ethikkodexen der Deutschen Gesellschaft für Soziologie (DGS), der Deutschen Gesellschaft für Psychologie e. V. (DGPs) und deren Berufsverbänden, der Deutschen Gesellschaft für Erziehungswissenschaft (DGfE) sowie des Deutschen Berufsverbandes für Soziale Arbeit e. V. (DBSH) liegt der Schwerpunkt auf Aspekten der *internen* Ethik [10–13]. In Abgrenzung zur *externen* Ethik, d. h. der Verantwortung gegenüber den Beforschten, wird hier die Verantwortung gegenüber der Wissenschaft und anderen Forschenden bzw. Beteiligten im wissenschaftlichen Feld (z. B. Studierenden) in den Blick genommen [1]. Dies gilt besonders für die Ethikkodexe der DGPs und der DBSH. In Bezug auf die Frage nach der Schadensminimierung werden zwar ungünstige Konsequenzen oder spezielle Risiken angesprochen, aber nicht weiter ausgeführt [11, 13]. Auch die DGPs sowie der DBSH konkretisieren diese allenfalls ansatzweise als „… z. B. potenzielle Risiken, Unbehagen oder mögliche anderweitige negative Auswirkungen, die über alltägliche Befindlichkeitsschwankungen hinausgehen“ [12, S. 23] bzw. als „Schutz der Klienten vor Verschlechterung ihrer Lage“ [10, S. 27].

Der Ethikkodex der Deutschen Gesellschaft für Soziale Arbeit (DGSA) geht dagegen auf die Verantwortung der Forschenden gegenüber den Beforschten sehr umfassend ein. Neben möglichen psychischen, körperlichen und zeitlichen Belastungen werden z. B. auch Enttäuschungen der Vertrauensbeziehung als Belastung genannt. Besonders hervorzuheben ist, dass der Ethikkodex auch die Einholung der Bewertung von Risiken und Belastungen aus Sicht der Forschungsteilnehmenden beinhaltet [14].

Zusammenfassend lässt sich konstatieren, dass in den Sozial- und Humanwissenschaften zwar grundsätzlich Einigkeit über ethische Prinzipien besteht, die auf die Beziehung zu den Menschen verweisen, die im Fokus der Forschung stehen. Der Schwerpunkt der Auseinandersetzung liegt aber häufig auf der Professionalität des jeweiligen Feldes und die Frage der Beziehung zu den Beforschten wird – abgesehen von den gesetzlichen Regelungen zum Datenschutz und der Anonymität – nicht im Detail diskutiert. Eine Ausnahme stellt hier der Ethikkodex der DGSA dar, der zudem für partizipative Forschung durchaus als einschlägig bewertet werden kann. Übereinstimmend werden ethische Fragen als prozessbegleitend angesehen [6, 9, 14], unabhängig davon, inwieweit die in der Praxis fortwährend auftretenden ethischen Fragestellungen tatsächlich ausgiebig reflektiert werden können.

### Ethische Anforderungen in der partizipativen Gesundheitsforschung

Partizipative Forschung ist in Deutschland noch wenig verbreitet, dementsprechend selten ist in diesem Forschungsbereich die Auseinandersetzung über ethische Fragestellungen. In der angloamerikanischen Literatur findet dagegen eine breitere Debatte statt, was auch durch stärker institutionalisierte Regelungen für die Sozialwissenschaften in Kanada, den USA und England begründet sein dürfte [6, 15].

Zunächst wird partizipative Forschung gegenüber nicht partizipativer Forschung als ethisch angemessener gesehen, als ein „inherently more ethical approach“ [16, S. 5]. Begründet wird dies u. a. durch die mit Partizipation einhergehende Demokratisierung von Forschung und Erkenntnisgewinn [17].

Daran anknüpfend sehen Flicker et al. in der Orientierung an den Grundprinzipien von partizipativer Forschung einen ethischen Kompass als Leitlinie für die Forschungspraxis. Zugleich erwachsen daraus jedoch in jeder Forschungsphase neue ethische Dilemmata [18, S. 228]. Hierfür wird von einigen Autor*innen der Begriff „everyday ethics“ [19], „everyday challenges“ [18] bzw. „applied ethical aspects“ [17] verwendet. Im Fokus der ethischen Auseinandersetzung stehen daher Fragen, die der Professionalität im jeweiligen Forschungsfeld zugeordnet werden können. Im Wesentlichen geht es um die Konsequenz, mit der Partizipation bzw. Entscheidungsteilhabe umgesetzt, und wie der Forschungsprozess gestaltet wird. Dieser beinhaltet die Gewinnung der Mitforschenden wie auch die Auswertung und Dissemination von Ergebnissen [16, 20]. Es geht also um die Umsetzung eines Ideals der Partizipation (die vollständige Gleichberechtigung) von in der Praxis sehr ungleichen Partner*innen bzw. um die Auseinandersetzungen mit der Frage, wie mit der inhärenten Ungleichheit in einem partizipativen Forschungsprojekt umgegangen werden kann. Scheinpartizipation (Tokenism) oder gar Instrumentalisierung sollen verhindert werden [21–23].

Auch für die partizipativen Forschungen werden die Prinzipien Freiwilligkeit/informierte Einwilligung, Schadensminimierung sowie Anonymisierung/Vertraulichkeit der Daten in einzelnen Publikationen angesprochen und zugleich kritisch diskutiert. Eine kritische Auseinandersetzung findet vor allem mit dem Prinzip der informierten Einwilligung sowie der Frage nach Vertraulichkeit und Anonymisierung statt. Die Frage nach der Schadensminimierung wird selten thematisiert, allerdings benennen einzelne Autor*innen das Problem der emotionalen Belastung der Forschenden aus der Lebenswelt im Zuge der Reflexion ihrer persönlichen Erfahrungen [16, 24, 25]. Überwiegend werden die für die nicht partizipative qualitative Forschung herangezogenen Prinzipien als nicht adäquat für partizipative Forschung angesehen [18, 24, 25].

Es versteht sich von selbst, dass für die partizipative Forschung ethische Kriterien vor allem prozessbezogen zu bearbeiten sind [18, 20, 25]. Wenn sich die ethische Auseinandersetzung vor allem auf Fragen der Umsetzung der Entscheidungsteilhabe in allen Phasen des Forschungsprozesses bezieht – von der Definition gemeinsamer Ziele bis hin zu Formaten für die Verbreitung von Ergebnissen –, können spezifische ethische Prinzipien aus dem Blickfeld geraten. Auch geht es dabei grundsätzlich um Fragen der Macht, deren Diskussion zwar zwingend erscheint, aber eine Auseinandersetzung mit spezifischen ethischen Kriterien nicht ersetzt. Hinzu kommt, dass beispielsweise die Intensität der Partizipation in der Regel durch die Wissenschaft definiert wird, welche sich wiederum nur selten an den Bedürfnissen der Forschenden aus der Lebenswelt orientiert [26].

Um diesem Dilemma zu begegnen, kann ein stärkerer Bezug auf die in der human- und sozialwissenschaftlichen Gesundheitsforschung anerkannten Prinzipien sinnvoll sein [27, 28]. Auch wenn die Fragen unterschiedlich gestellt werden, gibt es doch Schnittmengen in der Auseinandersetzung mit ethischen Herausforderungen.

Wir möchten deshalb die Erfahrungen eines Forschungsprojektes für eine Prozessreflexion nutzen, um aus ethischer Perspektive den Blick auf eine bislang wenig beachtete Frage zu lenken: Welche Beeinträchtigungen können die Forschenden aus der Lebenswelt in einem partizipativen Forschungsprojekt erfahren und was resultiert daraus für künftige Prozesse? Wir haben uns für die Verwendung des Begriffs „Beeinträchtigungen“ entschieden, weil die im Folgenden vorgenommene Reflexion weniger Schädigungen als vielmehr Beeinträchtigungen aufzeigt, aus denen ein Schaden resultieren kann. Wir haben außerdem bewusst die Perspektive der Forschenden aus der Lebenswelt gewählt, eine Ergänzung um die Perspektiven der übrigen Prozessbeteiligten war aufgrund des für diesen Beitrag gesetzten Rahmens nicht möglich.

## Ethische Herausforderungen der partizipativen Zusammenarbeit in der Praxis

Konzeptionelle Grundlage des Projektes *ElfE – Eltern fragen Eltern* war der Peerforschungsansatz. Eltern mit Kindern im Kitaalter haben zu der gemeinsam definierten Frage geforscht, wie die Zusammenarbeit zwischen Eltern und Kitafachkräften gestärkt werden kann (vgl. [29]). Ausgehend von dem *Partnermodell* nach Roche, Guta und Flicker sollten alle Beteiligten in gleichem Ausmaß auf den Forschungsprozess Einfluss nehmen können [30]. In der ersten Förderphase wurden Leitfadeninterviews mit anschließender Auswertung orientiert an sozialwissenschaftlichen Analyseverfahren mit 3 Forschungsteams durchgeführt (vgl. [31]). In der zweiten Förderphase folgte ein kompaktes, 8 Workshops umfassendes Format, das partizipative Fokusgruppen als Erhebungsmethode nutzte (vgl. [32]). Außerdem wurden die Wirkungen auf unterschiedliche Ebenen (z. B. beteiligte Eltern, kommunale Ebene) evaluiert.

Neben den Forschungsteams gab es eine Steuerungsgruppe, in der Akteure rund um das Setting Kita (v. a. die bezirkliche Fachsteuerung, der Bezirkselternausschuss Kita, ein Familienzentrum, eine Vertretung der Kitaträger sowie die Peerforschenden) eingebunden waren.

### Indikatoren für Beeinträchtigungen

In beiden Förderphasen wurden mit den forschenden Eltern schriftliche Arbeitsvereinbarungen abgeschlossen, in denen v. a. die Ziele des Forschungsprojektes, die Aufgaben sowie die Honorierung festgehalten waren. Als Indikator für Beeinträchtigungen waren darin emotionale Belastungen ausgelöst durch die Reflexion persönlicher Erfahrungen genannt. In beiden Förderphasen wurde deshalb ein regelmäßiges Feedback zum Ende der jeweiligen Arbeitstreffen implementiert. In Verbindung mit den Leitfadeninterviews wurde außerdem ein strukturiertes telefonisches *De-Briefing* ([30]; Nachbesprechung) durchgeführt^1^.

Ergänzend zu den emotionalen Belastungen wurden in der Arbeitsvereinbarung für die zweite Förderphase auch die Veränderungen der persönlichen Beziehungen im Lebensumfeld als Indikator genannt. Außerdem wurde eine Telefonliste mit (teils anonymen) Unterstützungsmöglichkeiten von den Eltern selbst erarbeitet.

Im Zuge der Evaluation der Wirkungen wurden in mehreren Workshops mit allen ElfE-Beteiligten die projektseitigen intendierten wie nicht intendierten Wirkungen erarbeitet und in einer Tabelle aufbereitet. Diese Wirkungen wurden nach Ebenen sortiert, so auch für die Ebene der teilnehmenden Eltern. Wir möchten die folgenden, von den Eltern definierten nicht intendierten Wirkungen zur Reflexion möglicher Beeinträchtigungen zugrunde legen:

1. Das persönliche soziale Netzwerk der beteiligten Eltern verringert sich.
2. Die Beziehung zu den Erzieher*innen ihrer Kinder verschlechtert sich.
3. Die beteiligten Eltern haben sich eine Fachsprache angeeignet. Es fehlt ihnen jetzt eine Sprache, um die Informationen an andere Eltern weiterzugeben.
4. Eltern fühlen sich von der Offenheit des Prozesses und dem Erwartungsdruck überfordert.
5. Die Eltern sind enttäuscht von partizipativer Forschung, weil sie keine Hilfe oder Lösung für ihre individuellen Probleme erfahren.

### Ergebnisse der Prozessreflexion zu möglichen Beeinträchtigungen

Die Frage nach den Wirkungen auf die forschenden Eltern wurde zu verschiedenen Zeitpunkten und auf verschiedene Weise im Prozessverlauf reflektiert: in Form von Leitfadeninterviews, die im Rahmen einer Masterarbeit durchgeführt wurden, im Zuge der routinemäßigen Reflexion in den Forschungsworkshops sowie in einer spezifischen Gruppendiskussion. Die jeweiligen Dokumentationen und auch die Masterarbeit wurden dann für eine Dokumentenanalyse (extern vergebener Honorarvertrag) genutzt.

In Bezug auf negative *Veränderungen des persönlichen Netzwerks* sowie der *Beziehung zu den Kitafachkräften* finden sich in den herangezogenen Dokumenten keine Belege. Im Gegenteil wird z. B. von neu entstandenen Freundschaften berichtet. Die *Veränderung der eigenen Sprache* (z. B. Verwendung kontextbezogener Fachbegriffe) wurde in der internen Diskussion bestätigt.

Hinweise auf mögliche *Überforderungen* fanden sich in Form von Irritationen darüber, dass Diskussionen teils als chaotisch erlebt wurden. Auch wurde die Moderation der Workshops durch einzelne Eltern von ihnen als belastend beschrieben und es wurden Tätigkeiten wie *Protokolle nachlesen *oder* Dokumente in den persönlichen Projektordner sortieren *als Herausforderungen genannt.

Als verunsichernd wurde die Beteiligung an anderen Arbeitsgremien (Steuerungsgruppe, Forschungsverbund) erlebt. Es fehle an Übung im Austausch von Argumenten, Strukturen und die damit verknüpften Handlungsmöglichkeiten seien nicht ausreichend bekannt, sodass ein Gefühl, nicht dazuzugehören, entstand.

*Enttäuschungen über das Ergebnis der Forschung* werden auf individueller und struktureller Ebene berichtet. Auch die ElfE-Eltern bestätigen den Willen, Veränderungen herbeizuführen, als wichtiges Beteiligungsmotiv [33, 34]. Deutlich wird das z. B. an dem Wunsch, durch das Forschungsteam Unterstützung für persönliche Gespräche in der Kita zu erhalten, was zwangsläufig enttäuscht werden musste. Auch die Wahrnehmung, keinen Einfluss auf aus den Forschungsergebnissen resultierende Veränderungen zu haben, löste Frustrationen aus. „Viel Engagement für nichts?“ oder „Was haben die Eltern am Ende von ElfE?“, waren diesbezüglich gestellte Fragen. Es zeigte sich an dieser Stelle eine deutliche Diskrepanz zwischen den Bedürfnissen der Eltern und den Zielen der Forschung, langfristig Einfluss auf die Lebensbedingungen zu nehmen.

### Reflexion der Ergebnisse zu möglichen Beeinträchtigungen durch die beteiligten Wissenschaftlerinnen^2^ sowie die Forschenden aus der Lebenswelt

Voranzustellen ist, dass die forschenden Eltern im Projekt ElfE in vielfältiger Weise von ihrer Mitwirkung profitiert haben. Unter anderem im Rahmen der zuvor angesprochenen Masterarbeit wurden Hinweise darauf gewonnen, dass das (intendierte) Ziel, *die Selbstvertretungskompetenz der beteiligten Eltern zu stärken, *erreicht wurde. Auch sind die Ergebnisse der Forschung in vielfältiger Weise in bezirkliche Angebote eingeflossen (vgl. Hilgenböcker et al. in diesem Themenheft).

Mit der Beteiligung einhergehende Risiken auf Beeinträchtigungen sind u. E. unvermeidbar und es bedarf eines bewussten Umgangs mit diesen Risiken. Die Ergebnisse der Prozessreflexion haben wir zunächst aus Sicht der Wissenschaft aufgearbeitet und dann mit den Forschenden aus der Lebenswelt gemeinsam überprüft, diskutiert und ergänzt. Auf dieser Basis können nachfolgende Schlussfolgerungen gezogen werden:

Auch wenn die Beteiligung keine direkten Auswirkungen auf das *Verhältnis zu den jeweiligen Kitafachkräften* hatte, wird aus Sicht der Eltern die Mitarbeit in einem wissenschaftlichen Forschungsprojekt als Kritik an den im Fokus stehenden Einrichtungen gesehen. Die forschenden Eltern haben daher ihre Projektbeteiligung nicht tiefergehend in ihrer Kita kommuniziert.

Ebenfalls hat sich die Veränderung der *Sprache *nicht auf das Verhältnis zu anderen Personen ausgewirkt, aber die Sprache an sich stellte eine fortlaufende Herausforderung dar. Obwohl Fachbegriffe möglichst vermieden bzw. (schriftlich wie mündlich) erläutert wurden, konnte sich z. B. selbst für den Begriff Partizipation ein Verständnis erst nach und nach entwickeln. Insgesamt wurde vor allem zu Beginn der Zusammenarbeit vieles nicht verstanden, teilweise aus Verunsicherung aber auch nicht nachgefragt.

In Bezug auf die mögliche *Überforderung* kamen die forschenden Eltern zu dem Schluss, dass nach ihrer Übernahme der Workshopmoderation die „Glückshormone und die Leichtigkeit“ dahin waren (vgl. [35]). Bei den Wissenschaftlerinnen hat dies einerseits die Frage nach der eigenen Verantwortung ausgelöst, die Eltern ggf. vor Überforderungen zu schützen. Andererseits waren sie selbst im Prozess immer wieder mit der Entscheidung überfordert, an welchen Aufgaben alle beteiligt werden sollten und wo es besser wäre, arbeitsteilig zu verfahren. Rückblickend bestätigen die Eltern die mit der Aufgabe verbundene Herausforderung (besonders wenn Personen dabei waren, die nicht zum Forschungsteam gehörten). Sie nahmen diese Herausforderung aber zugleich für ihre persönliche Weiterentwicklung bewusst an. Um die Herausforderung ohne Ängste meistern zu können, schlugen sie eine konkrete Unterstützung durch die Wissenschaft vor (z. B. gemeinsame Vor- und Nachbereitungen inkl. eines kritischen Feedbacks). Zugleich sollten auch die Überforderungen seitens der Wissenschaft transparent gemacht und für eine gemeinsame Diskussion genutzt werden.

Als (vermeidbare) Überforderung schätzen die Eltern dagegen den fehlenden Überblick über die verschiedenen Arbeitsstränge des Projektes ein. Es fehlte hier an Transparenz bzw. in verschiedener Hinsicht an Kontinuität, was zu Verunsicherungen geführt habe. Schriftliche Protokolle haben dies nicht aufgefangen, da diese zwar in einem personalisierten Projektordner zusammengefasst, aber nicht aktiv eingesetzt wurden.

Zu *Enttäuschungen* über das Ergebnis bzw. den Verlauf der Forschung kam es in Bezug auf die Balance zwischen dem Vorankommen im Prozess und der persönlichen Unterstützung der Eltern. Wie viel Raum wird für die gegenseitige Beratung über persönliche Erfahrungen gegeben? Dies verweist erneut auf die unterschiedlichen Interessen zwischen Wissenschaft und Praxis, wobei auch die Interessen der Forschenden untereinander variieren können. Der persönliche Austausch war für einzelne ElfE-Eltern durchgehend unzureichend, für andere nahm er zu viel Raum ein. Die beteiligten Wissenschaftlerinnen waren dagegen verantwortlich, das Projekt hinsichtlich von Output und Outcome weiterzubringen. Hinzu kommt eine nachlassende individuelle Relevanz, wenn die eigenen Kinder während des Prozesses aus dem Kitaalter herauswachsen.

Wir sehen hier den Bedarf nach einer kontinuierlichen Reflexion, dass nicht der persönliche, sondern der kollektive Nutzen im Vordergrund steht. Daraus leitet sich u. E. eine Entlohnung der Forschenden aus der Lebenswelt als ethisch geboten ab. Neben dem Argument der Fairness unterstützt der Erhalt eines Honorars, sich der Bedeutung des eigenen Beitrags bewusst zu werden, und grenzt diese von dem Erhalt einer persönlichen Hilfeleistung ab [36].

Der Einfluss auf die bezirklichen Strukturen brauchte Zeit und es galt, Vorbehalte zu überwinden. Dies hat zu *Enttäuschungen* geführt bzw. wurde als *Durststrecke* wahrgenommen. Die Eltern wünschen sich eine stärkere, Wiederholungen vermeidende Strukturierung der Zusammenarbeit. Für die Inspiration und Weiterentwicklung des Prozesses wurden die punktuell zugezogenen externen Sichtweisen als sehr bereichernd eingeschätzt.

## Diskussion

Aus unserer Sicht wäre zunächst zu reflektieren, ob die Forschung *mit *und nicht *über* Menschen per se das ethisch adäquatere Vorgehen ist. Diese Auseinandersetzung kann im Rahmen dieses Beitrags nicht vertieft werden. Ebenso wie in der nicht partizipativen Forschung lassen sich mindestens Beeinträchtigungen nicht vermeiden und es kann kein umfassender Schutz vor Schädigungen gewährleistet werden.

In dem illustrierten Forschungsprojekt wurden seitens der Wissenschaft mögliche Beeinträchtigungen zunächst vor allem als emotionale Belastungen angesehen, die durch die Reflexion persönlicher Erfahrungen ausgelöst werden. Die Forschenden aus der Lebenswelt haben wesentliche Indikatoren ergänzt, u. a. Herausforderungen wie die Sprache, Überforderungen durch die Komplexität des Projektes und Enttäuschungen in Bezug auf individuelle Ziele. Dies zeigt eindrücklich, wie gewinnbringend die partizipative Zusammenarbeit sein kann.

Das Beispiel macht zudem deutlich, dass es eines spezifischen Umgangs mit den ethischen Herausforderungen im partizipativen Forschungsprozess bedarf, für den die Wissenschaft/Projektverantwortlichen klar die Verantwortung haben. Folgende Hinweise können eine adäquate ethische Praxis unterstützen:

- Es bedarf einer Vorbereitung des *Feldes*, selbst wenn dieses (zunächst) nicht konkret beteiligt werden soll. Wenn bestehende Vorbehalte in eine Offenheit gewandelt werden können, unterstützt dies nicht nur den Zugang von Forschenden aus der Lebenswelt, sondern eröffnet auch Möglichkeiten für Rückkoppelungen und den Ergebnistransfer.
- Die Erarbeitung einer gemeinsamen, verständlichen Sprache hat sich als fortlaufende Herausforderung erwiesen. Es bedarf hier einer aktiven, prozessbegleitenden Nachfrage, die unterschiedliche Möglichkeiten der Unterstützung nutzt (Nachschlagemöglichkeiten, dialogische Verfahren). Die Bedeutung der Sprache sollte auch wegen ihrer Wirkung auf das Wohlbefinden und die Integration der Forschenden aus der Lebenswelt nicht unterschätzt werden.
- Der Umgang mit Herausforderungen benötigt eine Abwägung mit dem ggf. resultierenden persönlichen Gewinn. Als Fürsorge kann daher weniger deren Vermeidung als die gezielte Unterstützung im Umgang mit diesen gesehen werden.
- Das Bedürfnis, sich über persönliche Erfahrungen auszutauschen, kann kaum vollständig gemeinsam erfüllt werden. Stattdessen können separate Gespräche (ggf. mit externen Personen) angeboten werden.
- *Durststrecken* beeinträchtigen das Wohlbefinden, verweisen u. E. zugleich auf notwendige (Neu‑)Orientierungen im Prozess und können Ausgangspunkt für Weiterentwicklungen sein. Sie lassen sich durch eine Strukturierung und Steuerung auch im partizipativen Prozess eingrenzen. Es braucht eine Balance, *Räume zu geben vs. Wiederholungen zu vermeiden*. Die Einbeziehung weiterer Perspektiven kann wichtiger Impuls für Weiterentwicklungen sein.
- Aufwandsentschädigungen für die Forschenden aus der Lebenswelt tragen dem kollektiven Interesse an den Ergebnissen der Forschung Rechnung.
- Für die Forschenden aus der Lebenswelt ist es wichtig, alle Aktivitäten des Forschungsprojektes zu kennen, auch wenn sie nicht selbst daran mitwirken. Es besteht durchaus Interesse an der wissenschaftlichen Verwertung, sofern diese nachvollziehbar ist.

Insgesamt resultiert aus den abgeleiteten Hinweisen ein Bedarf an Raum und Zeit für gemeinsame und kontinuierliche Reflexion. Neben vorbereitenden Aktivitäten ergeben sich daraus zusätzliche Aufgaben im Prozess. Auch ist eine Balance zwischen den Interessen der Forschenden aus der Lebenswelt und der Wissenschaft zu finden. Ein ausreichender Zeitrahmen für jedes gemeinsame Arbeitstreffen ist dafür essenziell.

## Fazit

Partizipative Forschung verläuft in der Regel dynamisch und ethische Fragen können dabei leicht in den Hintergrund geraten. Am hier zugrunde gelegten Prinzip der Schadensminimierung wird deutlich, dass eine Orientierung an den ethischen Prinzipien der qualitativen Forschung (an der sich partizipative Forschung auch im methodischen Vorgehen orientiert) adäquat sein kann. Zugleich wird illustriert, dass sich die konkreten Fragen gegenüber der nicht partizipativen Forschung anders stellen und zudem herausfordernder sind.

Die Einbeziehung der Perspektive der Forschenden aus der Lebenswelt hat das Verständnis über mögliche Beeinträchtigungen im partizipativen Forschungsprozess erweitert. Auch wenn vom Kontext abhängig, sind diese u. E. für die partizipative Zusammenarbeit typisch.

Die Begrenzung auf dieses bislang in der partizipativen Forschung wenig beachtete Prinzip war für die tiefergehende Auseinandersetzung notwendig. Das Prinzip steht zugleich exemplarisch für den Bedarf, eine umfassende ethische Betrachtung aktiv in den Prozess zu integrieren. Dafür kann es sinnvoll sein, in der Planungsphase konzeptionell festzulegen, anhand welcher ethischer Prinzipien und Kriterien die Reflexion erfolgen soll und wie die Perspektive der Forschenden aus der Lebenswelt integriert wird. In diesem Rahmen sollten die Forschenden aus der Lebenswelt regelmäßig erinnert werden, dass ein Schutz vor Beeinträchtigungen niemals umfassend gewährleistet werden kann.

Die abgeleiteten Hinweise wirken sich auch auf die Gestaltung des Prozesses aus. Ein strukturiertes bzw. steuerndes Vorgehen muss nicht im Widerspruch zu gleichberechtigter Entscheidungsteilhabe stehen, es kann Enttäuschungen vermeiden und so die Motivation stärken. Transparenz über die Projektaktivitäten und Klarheit über die Rahmenbedingungen können das Wohlbefinden der Forschenden aus der Lebenswelt stärken.

Nicht zuletzt wünschen wir uns, mit dem Beitrag die inhaltliche wie prozessuale Auseinandersetzung mit ethischen Aspekten in der partizipativen Forschung zu stärken. Der Ethikkodex der Deutschen Gesellschaft für Soziale Arbeit kann dafür ein geeigneter Anknüpfungspunkt sein. Er führt die Verantwortung gegenüber den Forschungsteilnehmenden vergleichsweise umfassend aus und ist aus unserer Sicht insgesamt an die partizipative Gesundheitsforschung gut anschlussfähig.

## Footnotes

1 Erfragt wurde u. a.: „Wie war der Verlauf?“ „Welche Themen haben dich besonders beschäftigt?“ „Welche ergänzenden Fragen hast du?“

2 In dem betreffenden Forschungsprojekt waren ausschließlich Wissenschaftlerinnen tätig, sodass wir auf die männliche Form verzichten.
